# Elevated adipokines and myokines are associated with fatigue in long COVID patients

**DOI:** 10.3389/fmed.2025.1547886

**Published:** 2025-05-19

**Authors:** Nina R. G. Visconti, Nazareth N. Rocha, Gabriela S. Nascimento, Caio V. B. Menário, Johnatas D. Silva, Camila M. Martins, Celso Caruso-Neves, Fernanda F. Cruz, Patricia R. M. Rocco, Fernanda C. Q. Mello, José R. Lapa-e-Silva

**Affiliations:** ^1^Thoracic Disease Institute, Federal University of Rio de Janeiro, Rio de Janeiro, Brazil; ^2^Carlos Chagas Filho Institute of Biophysics, Federal University of Rio de Janeiro, Rio de Janeiro, Brazil; ^3^Biomedical Institute, Fluminense Federal University, Niterói, Brazil; ^4^Department of Medicine, State University of Ponta Grossa, Ponta Grossa, Brazil

**Keywords:** post-COVID-19, fatigue, myokines, adipokines, lung function, ultrasonography

## Abstract

**Background:**

Persistent fatigue is one of the most common and debilitating symptoms experienced by patients recovering from COVID-19, contributing significantly to the burden of “long COVID” or post-COVID-19syndrome. However, the underlying pathophysiological mechanisms remain inadequately understood. Few studies have examined the association between fatigue and pulmonary or cardiac function, systemic biomarkers, or morphological changes in the lungs and diaphragm. Furthermore, the potential influence of vaccination on the persistence of fatigue has not been fully explored. This study aims to identify mechanisms contributing to post-COVID-19 fatigue.

**Methods:**

This prospective cohort study assessed clinical, laboratory, pulmonary, and cardiac parameters, as well as diaphragm ultrasound and pulmonary function, in patients with and without fatigue at least 4 months after discharge from hospitalization due to COVID-19.

**Results:**

Of 88 patients evaluated, 34% reported new or worsening fatigue after recovering from COVID-19. Demographic characteristics, comorbidities, and vaccination status were similar between fatigued and non-fatigued groups. However, ICU admission during the acute phase of illness emerged as a significant risk factor for fatigue (OR 2.65; 95% CI, 1.03–6.94) in multivariable analysis. No significant differences were observed in lung function, diaphragm or lung ultrasound findings, or left ventricular systolic function between groups. Fatigue was associated with significantly elevated serum levels of myostatin and irisin, markers of muscle metabolism. Additionally, patients experiencing fatigue reported poorer functional capacity and significantly reduced quality of life, with lower scores in multiple domains of the SF-36 questionnaire, including general health, vitality, and mental health.

**Conclusion:**

Post-COVID-19 fatigue is strongly associated with prior ICU admission and elevated levels of myostatin and irisin, implicating potential myopathic mechanisms in its persistence. The profound impact of fatigue on functional capacity and quality of life highlights the urgent need for further research to elucidate its pathophysiology and develop targeted therapeutic strategies. This study provides critical insights into the interplay between systemic and organ-specific factors contributing to fatigue, offering a foundation for future interventions to improve outcomes in patients with long COVID.

## 1 Introduction

Despite significant advancements in understanding and managing COVID-19, the long-term health consequences of this disease remain a major concern, even five years after the onset of the SARS-CoV-2 pandemic (1). A substantial proportion of individuals experience persistent symptoms beyond the acute phase of infection, collectively termed “long COVID” or the “post-acute COVID-19 syndrome.” This condition is characterized by the appearance or persistence of symptoms at least three months after the initial infection and lasting for a minimum of two months without an alternative explanation. Long COVID has affected an estimated 65 million people worldwide, with incidence rates varying widely: 10–30% among non-hospitalized cases, 50–70% among hospitalized patients, and 10–12% in vaccinated individuals (2).

Among the myriad symptoms reported, fatigue emerges as one of the most prevalent and disabling manifestations of long COVID, with studies estimating its prevalence at 28% even two years post-infection (3–7). This symptom profoundly impacts patients’ quality of life, yet its pathophysiological mechanisms remain poorly understood. Proposed contributors include immune dysregulation, autonomic dysfunction, and metabolic alterations. Some researchers have drawn parallels between post-COVID-19 fatigue and chronic fatigue syndrome (8), although their shared mechanisms remain speculative (9).

To address this gap, the present study aims to identify mechanisms contributing to post-COVID-19 fatigue by investigating pulmonary and cardiac function and potential morphological changes in the lungs and diaphragm in patients with and without long COVID. We hypothesize that post-COVID fatigue is associated with prior ICU admission and is linked to alterations in muscle-related biomarkers, specifically higher levels of myostatin and irisin. Our study suggests that systemic factors related to critical illness, such as prolonged ICU stay, contribute to persistent muscle dysfunction, which is reflected in elevated myostatin and irisin levels. These molecular changes may emphasize the chronic fatigue experienced by post-COVID patients, highlighting a potential mechanistic pathway involving muscle metabolism dysregulation. Furthermore, the potential influence of vaccination on the persistence of fatigue was explored.

## 2 Methods

### 2.1 Study design and setting

This prospective cohort study was conducted at Clementino Fraga Filho University Hospital (CFFUH), Federal University of Rio de Janeiro, Brazil. Patients hospitalized with acute COVID-19 between March 2020 and May 2022 were recruited for follow-up at the CFFUH outpatient clinic via telephone. COVID-19 was confirmed by a positive PCR test for SARS-CoV-2. Follow-up assessments occurred at least four months post-hospital discharge. The study protocol was approved by the local Research Ethics Committee (CAAE: 53517521600005257).

### 2.2 Procedures

#### 2.2.1 Data collection

Baseline and clinical data from the acute phase were extracted from medical records, with disease severity classified as per World Health Organization guidelines (10). At follow-up, symptoms were systematically accessed via structured questionnaires (Supplementary Data Sheet 1) and quality of life was evaluated using the Short-Form Health Survey 36 (SF-36). Symptoms were defined as “present” if they appeared after COVID-19 or worsened following infection.

#### 2.2.2 Laboratory analysis

Blood samples were collected for routine biochemical and hematological evaluations and biomarkers (myostatin, irisin, leptin, and adiponectin) were quantified using commercial ELISA kits (Abcam, USA). Serum concentrations were determined from standard curves and normalized to protein content using the Bradford method. Hemoglobin, creatinine, and D-dimer levels were measured to assess anemia, renal function, and thrombotic risk.

#### 2.2.3 Lung function testing

Comprehensive pulmonary function testing (PFT) was performed with Master Screen Body and impulse oscillometry systems (Vyaire Medical GmbH, Germany), adhering to American Thoracic Society and European Respiratory Society guidelines (11–15). Obstruction was defined as FEV1/FVC below the lower limit of normal (LLN) (16, 17), restriction as total lung capacity (TLC) below the LLN (18), and DLCO abnormalities as values below the LLN after hemoglobin correction. Small airway disease (SAD) was identified by impulse oscillometry criteria (ΔR5-20 > 35% or X5 ≥ 0.15 kPa.s.L^−1^) (19).

#### 2.2.4 Ultrasound

Ultrasound assessments of the lungs, diaphragm, and heart were performed using a Versana Active device equipped with high-frequency linear (L6-12) and phased array (3 Sc-Rs) probes (GE HealthCare, Contagem, Brazil). Lung ultrasound scanning (LUS) was performed as described by Bouhemad et al. (20), with LUS scores categorized by severity as none (0), mild (1–5), moderate (6–14, 21), and severe (15–20, 22–36).

Diaphragmatic sonography was performed using a 3.5–5 MHz phased array probe and a 6.0–12 MHz high-frequency linear probe to measure diaphragm excursion and thickening fraction. For excursion measurements, the probe was placed below the right costal margin along the mid-clavicular line, and M-mode was used to measure diaphragmatic displacement in millimeters. Diaphragm thickness was assessed at the zone of apposition to the rib cage during quiet breathing and maximal inspiratory/expiratory effort. The thickening fraction (TF) was calculated as TF = (thickness at end-inspiration – thickness at end-expiration)/(thickness at end-expiration) (23). The left ventricular ejection fraction (LVEF) was measured using the biplane method of disks (modified Simpson method) (24).

## 3 Statistical analysis

Descriptive statistics included the mean, median, standard deviation, and interquartile range for quantitative variables, as well as absolute and relative frequencies for qualitative variables. The chi-square test was used to assess associations between qualitative variables. The Kolmogorov–Smirnov test with Lilliefors’ correction was used to evaluate the normality of the data, while the Levene median test was applied to assess the homogeneity of variances. Variables related to lung function, lung and diaphragm ultrasound, the SF-36 questionnaire, and biomarkers were compared using the Mann–Whitney U test, whereas age and BMI were compared using Student’s t-test. Laboratory parameters and SF-36 scores were compared over time and in relation to post-COVID fatigue using graphs and the Mann–Whitney test. A correlogram illustrating correlations between laboratory parameters and SF-36 variables was generated using Spearman’s correlation test. As a multivariate approach, cluster analysis was used to identify groupings of symptoms and associated sociodemographic and clinical conditions, as well as their relationship with the presence of fatigue. The agglomerative hierarchical method was employed, where the number of clusters is determined based on the grouping generated by the analysis itself. Each category of variables was transformed into dummy variables to enable cluster analysis for binary data. In this case, the simple matching method was used to calculate the similarity (distance) measure between variables. For the chaining of observations, the average linkage method was applied, which merges groups based on the average distance between all pairs of observations within the groups under analysis. The determination of clusters begins with the number of clusters equal to the number of observations, and then two observations with the smallest distance are merged. This process continues until a single cluster is formed. Cluster formation is visualized on a dendrogram. All analyses were performed in the R 4.1.0 software environment (50).

### 3.1 Results

Among 1,535 COVID-19 patients admitted to Clementino Fraga Filho University Hospital, 990 were discharged and 99 attended the post-COVID-19 outpatient clinic. Of these, 88 completed evaluations at least four months after discharge. Fatigue was reported by 34% (30 patients) during follow-up. Baseline characteristics, including comorbidities, gender, and age, showed no significant differences between patients with and without fatigue, as detailed in Table 1.

**Table 1:** Descriptive statistics and comparison of patients with and without post-COVID-19 fatigue during follow-up.

		Post-COVID-19 fatigue				
Variables		All patients (*n* = 88)	Yes *N* (lin%)	No *N* (lin%)	OR (95%CI)	*p*-value*
Timing of evaluation	< 1 year post COVID	46 (52)	13 (28)	33 (72)	REF	
	1–2 years post COVID	24 (27)	12 (50)	12 (50)	2.54 (0.91–7.08)	0.072
	> 2 years post COVID	18 (20)	5 (28)	13 (72)	0.98 (0.29–3.29)	0.969
Gender	Male	36 (41)	9 (25)	27 (75)	REF	0.134
	Female	52 (59)	21 (40)	31 (60)	2.03 (0.78–5.18)	
Age (years)	< 60 years	49 (56)	19 (39)	30 (61)	REF	0.416
	≥ 60 years	39 (44)	11 (28)	28 (72)	0.62 (0.25–1.53)	
BMI (kg/m^2^)	< 30	51 (61)	16 (31)	35 (69)	REF	
	> 30	33 (39)	13 (39)	20 (61)	1.42 (0.56–3.55)	0.603
Vaccination status	Unvaccinated (%)	38 (44)	14 (37)	24 (63)	REF	
	One or more doses	49 (56)	16 (33)	33 (67)	0.83 (0.34–2.02)	0.857
Comorbidities	Yes	83 (94)	28 (34)	55 (66)	0.76 (0.12–4.84)	1.000
Arterial hypertension	Yes	52 (59)	16 (30)	36 (70)	0.70 (0.29–1.70)	0.575
Diabetes mellitus	Yes	25 (28)	10 (40)	15 (60)	1.43 (0.54–3.74)	0.626
Asthma	Yes	7 (8)	5 (71)	2 (29)	5.60 (1.02–30.85)	0.079
COPD	Yes	4 (5)	2 (50)	2 (50)	2.00 (0.27–1.95)	0.883
Neoplasia	Yes	18 (20)	5 (28)	13 (72)	0.69 (0.22–2.17)	0.723
Interstitial lung disease	Yes	3 (3)	0 (0)	3 (100)	0.00	0.517
Prior transplantation	Yes	10 (11)	5 (50)	5 (50)	2.12 (0.56–8.00)	0.440
Auto immune disease	Yes	6 (7)	2 (33)	4 (67)	0.96 (0.17–5.59)	1.000
Chronic kidney disease	Yes	12 (14)	3 (25)	9 (75)	0.60 (0.15–2.43)	0.699
Ever smoker	Yes	28 (32)	12 (43)	16 (57)	1.75 (0.69–4.44)	0.236

**Chi-square or Pearson test as appropriate; *N*, absolute frequency; lin%, relative frequency in factors analyzed in each line; OR, odds ratio; CI, confidence interval; REF, reference category used in odds ratio.

A total of 52% of patients were evaluated within one year after discharge (original infection during the predominance of the Omicron variant in Rio de Janeiro), while 27% were assessed between one-and two-years post-discharge (original infection coinciding with the Delta variant), and 20% were evaluated more than two years after discharge (reflecting original infection during the Wuhan strain period). The prevalence of fatigue did not vary significantly based on the timing of the evaluations.

Vaccination status was comparable between the two groups, with 56% of patients having received at least one vaccine dose prior to their acute infection. Statistical analysis revealed no significant difference in vaccination status between fatigued and non-fatigued patients (*p* = 0.857, Table 1).

Hospitalization variables during the acute phase of COVID-19, including the need for oxygen supplementation, ICU admission, non-invasive ventilation, mechanical ventilation, and corticosteroid use, did not differ significantly between the groups (Table 2). However, ICU admission was associated with a marginally higher prevalence of fatigue in univariate analysis (*p* = 0.073), and multivariable logistic regression confirmed it as the sole independent risk factor for persistent fatigue (OR 2.65; 95% CI: 1.03–6.94). Similarly, a trend toward increased fatigue prevalence was observed among patients classified as critical cases by WHO guidelines (*p* = 0.051).

**Table 2:** Descriptive statistics in overall population and comparison between patients with and without post-COVID-19 fatigue, stratified by hospitalization-related variables.

Variables		Post-COVID-19 fatigue				
		All patients *N* (tot%)	Yes *N* (lin%)	No *N* (lin%)	OR (CI 95%)	*p*-value*
O_2_supplementation	No	50 (57)	14 (28)	36 (72)	REF	0.248
	Yes	38 (43)	16 (42)	22 (58)	1.87 (0.77–4.56)	
Admission to Intensive Care Unit	No	62 (70)	17 (27)	45 (73)	REF	0.073
	Yes	26 (30)	13 (50)	13 (50)	2.65 (1.02–6.84)	
Non-invasive ventilation	No	85 (97)	29 (34)	56 (66)	REF	1.000
	Yes	3 (3)	1 (33)	2 (67)	0.97 (0.84–11.10)	
Mechanical ventilation	No	80 (91)	25 (31)	55 (69)	REF	0.166
	Yes	8 (9)	5 (62)	3 (38)	3.67 (0.81–16.56)	
Corticosteroids	No	48 (55)	13 (27)	35 (73)	REF	0.196
	Yes	40 (45)	17 (43)	23 (57)	1.99 (0.81–4.86)	
Case severity classification (WHO)	Mild	44 (50)	12 (27)	32 (73)	REF	
	Moderate	6 (7)	2 (33)	4 (67)	1.33 (0.22–8.25)	0.756
	Severe	30 (34)	11 (37)	19 (63)	1.54 (0.57–4.18)	0.391
	Critical	8 (9)	5 (62)	3 (38)	4.44 (0.92–21.53)	0.051

**Chi-square or Pearson test as appropriate; *N*, absolute frequency; tot%, relative frequency in total of patients; lin%, relative frequency in factors analyzed in each line; OR, odds ratio; CI, confidence interval; REF, reference category used in odds ratio.

Pulmonary function tests revealed that 20% of patients exhibited an obstructive ventilatory pattern, 12% a restrictive pattern, 39% reduced diffusing capacity for carbon monoxide (DLCO), and 32% had SAD identified via impulse oscillometry (Supplementary Table 1). However, the prevalence of these ventilatory disorders and the median values of analyzed parameters were similar between the two groups (Table 3). Results from the SF-36 questionnaire indicated significantly lower scores in fatigued patients across various domains, including functional capacity, general health, vitality, social functioning, emotional aspects, and mental health (*p* < 0.05; Supplementary Table 2).

**Table 3:** Descriptive statistics in overall population and comparison between patients with and without post-COVID-19 fatigue stratified by lung function abnormalities and ultrasound findings.

Variables		Post-COVID-19 fatigue				
		All patients *N* (tot%)	Yes *N* (lin%)	No *N* (lin%)	OR (CI 95%)	*p*-value*
Spirometry	No obstruction	61 (80)	21 (34)	40 (65)	REF	1.000
	Obstruction	15 (20)	5 (33)	10 (66)	0.95 (0.29–3.15)	
Plethysmography	No restriction	53 (88)	21 (39)	32 (60)	REF	0.373
	Restriction	7 (12)	1 (14)	6 (86)	0.25 (0.03–2.26)	
DLCO	Normal	44 (61)	17 (39)	27 (61)	REF	
	Reduced	28 (39)	7 (25)	21 (75)	0.53 (0.19–1.51)	0.868
Impulse oscillometry	Normal	51 (68)	18 (35)	33 (65)	REF	
	Small airway disease	24 (32)	8 (33)	16 (67)	0.92 (0.33–2.55)	1.000
Pleural thickening	No	79 (89)	27 (34)	52 (65)	REF	1.000
	Yes	8 (9)	3 (38)	5 (62)	1.16 (0.26–5.20)	
Pulmonary consolidation	No	79 (89)	28 (35)	51 (64)	REF	0.840
	Yes	8 (9)	2 (25)	6 (75)	0.61 (0.11–3.21)	
≥ 3 B-lines	No	70 (79)	47 (67)	23 (32)	REF	0.717
	Yes	17 (20)	14 (31)	31 (69)	1.43 (0.48–4.24)	
Lung score	Mild	14 (16)	4 (29)	10 (71)	REF	0.887
	Moderate	9 (10)	3 (33)	6 (67)	1.25 (0.21–7.61)	
	Normal	65 (74)	23 (35)	42 (65)	1.37 (0.39–4.86)	
Diaphragm excursion amplitude	Normal	57 (65)	18 (32)	39 (68)	REF	0.661
	Reduced	31 (35)	12 (39)	19 (61)	1.37 (0.55–3.41)	
Diaphragm thickening fraction	Normal	69 (78)	25 (36)	44 (64)	REF	0.419
	Diaphragmatic dysfunction	19 (22)	5 (26)	14 (74)	0.63 (0.20–1.95)	

*Chi-square or Pearson test as appropriate; *N*, absolute frequency; tot%, relative frequency in total of patients; lin%, relative frequency in factors analyzed in each line; OR, odds ratio; CI, confidence interval; REF, reference category used in odds ratio; DLCO, carbon monoxide diffusing capacity; IOS, impulse oscillometry system.

Diaphragm ultrasound showed a reduced excursion amplitude in 35% of patients and impaired thickening fraction in 22%, with no significant differences between fatigued and non-fatigued patients. Lung ultrasound findings, including B-lines (20%), pulmonary consolidation (9%), and pleural thickening (9%), also showed no significant variation between the groups. Most patients had a normal lung score (74%), while 16% exhibited mild and 10% moderate abnormalities (Table 3).

Cardiac function analysis identified no instances of moderate-to-severe left ventricular dysfunction among fatigued patients, while one non-fatigued patient exhibited this condition, showing no classical signs of cardiac decompensation.

For laboratory parameters, there was significant difference of irisin, myostatin, leptin and D-dimer between patients with and without post-COVID fatigue (*p* < 0.05 – Table 4) and this was similar independently of time of evaluation (Supplementary Figures 1A,B). Multivariable logistic regression identified only myostatin (OR 2.14; 95% CI: 1.51–3.97) and irisin (OR 1.01; 95% CI: 1.01–1.02) as independent factors significantly associated with fatigue.

**Table 4:** Descriptive statistics in overall population and comparison between patients with and without post-COVID-19 fatigue, stratified by blood test results and biomarkers.

Variables	Post-COVID-19 fatigue			
	All patients MD (IQR)	Yes MD (IQR)	No MD (IQR)	*p*-value*
Hemoglobin (g/dL)	13.2 (11.8–14.2)	13.2 (11.6–14.2)	13.2 (11.9–14.2)	0.934
CRP (mg/L)	0.7 (0.6–1.6)	5.7 (1.9–8.9)	0.6 (0.5–0.7)	0.717
CPK total (U/L)	268.8 (204.4–372.7)	386.8 (255.8–520.2)	251.2 (199.3–306.2)	0.544
D-dimer (ng/mL)	6.1 (5.2–7.4)	5.8 (4.7–6.8)	6.2 (5.3–7.7)	0.028
Creatinine (mg/dl)	1.6 (1.0–2.2)	0.9 (0.8–1.1)	1.8 (1.4–3.4)	0.757
Ferritin (ng/mL)	3.5 (2.0–7.3)	4.4 (1.5–8.0)	3.4 (2.3–6.1)	0.067
Myostatin (pg/mg)	79.5 (57.0–114.7)	93.0 (69.0–106.0)	74.0 (55.0–120.5)	<0.001
Irisin (pg/mg)	625.5 (428.5–1256.2)	789.0 (579.2–1986.5)	536.5 (385.0–994.5)	0.001
Adiponectin (pg/mg)	1.0 (0.8–1.5)	1.1 (0.8–1.6)	1.0 (0.8–1.5)	0.071
Leptin (pg/mg)	123 (63.7–297.0)	111.0 (59.5–169.5)	180.0 (70.0–332-0)	<0.001

*Mann–Whitney U test; MD, Median; IQR, interquartile range.

For SF-36, there was significant difference of functional capacity, general health, vitality, emotional and social aspects, and mental health between patients with and without post-COVID fatigue (*p* < 0.05; Supplementary Table 2) and in patients evaluated less than 1 year after acute infection this was more evident (Supplementary Figure 2). When separated by time of evaluation, in general, for patients evaluated more than 2 years after acute infection these differences were greater than that evaluated less than 1 year (Supplementary Figure 2).

Correlations between important laboratory parameters and SF-36 variables are presented in Supplementary Figures 3–5. There was no strong correlation between them for all population study or when separated by with and without post-COVID fatigue.

**Figure 1:**
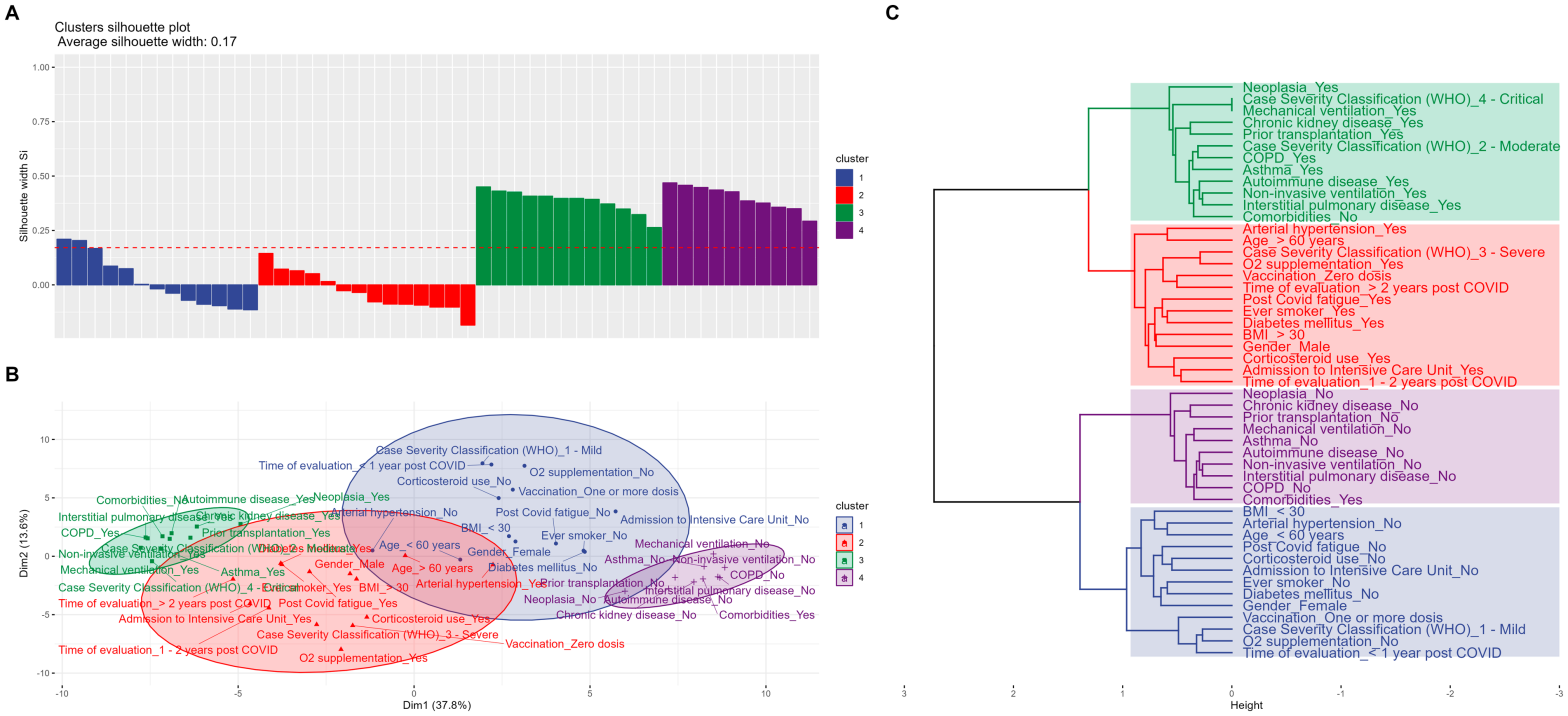
Multivariate analysis using the agglomerative hierarchical clustering method. Variables and categories sharing the same color indicate a strong association. **(A)** Average silhouette score for each cluster, illustrating the clustering quality. **(B)** Dendrogram depicting relationships and associations among variables. **(C)** Correspondence plot representing variables and clustering dimensions along the x-and y-axis.

Four variable clusters were identified (Figure 1). In the cluster represented by the green color, the profile found that patients with neoplasms, moderate or critical severity classification in the acute phase according to the WHO criteria, transplant recipients, those with renal diseases, COPD, asthma, autoimmune diseases and/or interstitial lung disease, and those who required mechanical or non-invasive ventilation were strongly associated with each other, but with no association with fatigue. In the cluster represented by the red color, the profile found was that those patients with hypertension, over 60 years old, WHO classification 3, unvaccinated, smokers, with diabetes, obesity, males, and those who received corticosteroids in the acute phase were strongly associated with each other and with the presence of fatigue. In the cluster represented by the purple color, only patients without the most severe symptoms (identified in the green cluster) were strongly associated with each other. In the cluster represented by the blue color, patients without the factors of the red cluster, associated with each other and with the absence of fatigue, were strongly linked to each other. Among the four clusters, those most representative of the patient profile included in the study were the green and purple clusters (Figure 1A).

## 4 Discussion

Post-COVID-19 fatigue remains one of the most debilitating and poorly understood consequences of SARS-CoV-2 infection, significantly affecting patients’ quality of life. While previous studies have documented its persistence (25), the underlying mechanisms remain unclear. This study provides novel insights by establishing a direct association between prior ICU admission, alterations in muscle-related biomarkers, and the persistence of fatigue in post-COVID patients. A key finding is the identification of elevated myostatin and irisin levels as potential molecular markers of post-COVID fatigue, highlighting a possible mechanistic pathway.

Baseline characteristics, including comorbidities, age, and gender, did not significantly differ between patients with and without fatigue. Notably, the prevalence of fatigue remained consistent across different time intervals post-discharge, regardless of whether evaluations occurred within one year or beyond two years. Vaccination status was also not significantly associated with fatigue, contrasting with previous studies suggesting a protective effect of vaccination against post-acute COVID-19 syndrome (26–29). One potential explanation is that a significant portion of the study population received only a single vaccine dose, which may have attenuated the protective effect, as prior research demonstrates greater efficacy with two doses (28).

This study’s longitudinal design, incorporating detailed clinical assessments across various infection waves, including those dominated by the Delta and Omicron variants, provides valuable insights into how evolving viral strains influence long-term outcomes. The inclusion of diverse evaluations, such as pulmonary function tests, diaphragm and lung ultrasound, and laboratory tests, enhances the robustness of the findings by offering a comprehensive view of post-infection health status.

Our findings align with existing literature highlighting persistent fatigue as a common symptom among COVID-19 survivors, affecting approximately one-third of patients months after infection (7, 30, 31). Hospitalization-related variables, including oxygen supplementation, mechanical ventilation, and severity classification, did not differ significantly between fatigued and non-fatigued groups. However, ICU admission emerged as a significant risk factor for persistent fatigue in multivariable analysis. The relationship between post-COVID-19 fatigue and the severity of the acute phase of the disease remains inconsistent in the literature (31). While some studies have reported a positive correlation (29, 32, 33), others did not identify any such association (5, 31, 34). Variability in access to intensive care units, differences in treatment protocols, and disparities in the quality of supportive care across centers may contribute to these discrepancies. In our study, the positive association between ICU admission and fatigue suggests that the severity of the initial illness may influence recovery trajectories. This aligns with evidence indicating that critical illness often results in prolonged physical and mental health challenges, including the post-intensive care syndrome (PICS) (35).Notably, we did not observe a significant relationship between ventilatory abnormalities and persistent fatigue, which is consistent with previous reports (36, 37). The prevalence of obstructive, restrictive, and small airway disease patterns observed in our cohort aligns with existing studies on post-acute sequelae of COVID-19, suggesting that pulmonary abnormalities may persist independently of subjective fatigue symptoms (37–40).

Unlike transient fatigue, post-COVID fatigue (41) is characterized by its chronicity and profound impact on both physical and mental well-being, often persisting for months after the resolution of the primary illness. Fatigue is a multidimensional symptom influenced by various factors, including physical deconditioning, psychological stress, and systemic inflammation. However, its precise etiology in post-COVID syndrome remains poorly understood, necessitating further research into its underlying pathophysiology. Given its complexity, post-COVID fatigue should be carefully distinguished from other conditions, such as chronic fatigue syndrome, depression, sleep disorders, and other post-viral fatigue syndromes. A thorough clinical evaluation is essential to rule out these potential diagnoses before attributing fatigue to post-COVID syndrome (42). Several tools are available to assess fatigue, each with its own advantages and limitations. Commonly used measures include the Fatigue Severity Scale, the Short Form-36 Health Survey, the Visual Analog Scale for Fatigue, the Chalder Fatigue Scale, and polysomnography for assessing sleep disorders (42, 43). However, further studies are needed to develop and validate assessment tools specifically tailored to the unique characteristics of post-COVID fatigue.

In the context of muscle-related biomarkers, elevated levels of myostatin and irisin were observed in patients experiencing fatigue, providing novel insights into the potential mechanisms underlying post-COVID-19 fatigue. Myostatin, a well-established negative regulator of muscle growth (44–46) has been implicated in muscle wasting and functional decline, particularly in critically ill patients. Its elevated levels in post-COVID patients, especially those who required ICU admission, suggest prolonged muscle dysfunction as a contributing factor to persistent fatigue. Additionally, previous studies have associated increased myostatin levels to prolonged hospital stays during COVID-19, likely due to reduced physical activity during acute infection. This further supports the role of myostatin in post-COVID muscle impairment and highlights its potential as a biomarker for identifying individuals at risk of long-term fatigue and functional decline.

Similarly, irisin, a myokine involved in energy metabolism and muscle adaptation (47, 48), has been associated with metabolic regulation. The observed increase in irisin levels may represent a compensatory response to counteract muscle deterioration. However, its sustained elevation could indicate an ongoing dysregulated metabolic state, suggesting a persistent imbalance in muscle homeostasis in post-COVID patients. Irisin also has potential anti-inflammatory effects. Shao et al. proposed that irisin modulates macrophage activity by reducing overproduction of reactive oxygen species (ROS), potentially mitigating lung injury. Oliveira et al. further demonstrated that irisin influences expression of genes associated with COVID-19 outcomes in adipose tissue. Specifically, it reduces the expression of genes such as *FURIN* and *ADAM10*, which are associated with increased viral replication, while upregulating *TRIB3*, which inhibits viral proliferation. These findings suggest the beneficial effects of irisin extend beyond anti-inflammatory actions to include modulation of viral replication mechanisms (49). In summary, our findings suggest that post-COVID fatigue may involve complex interactions in muscle metabolism and highlight myostatin and irisin as potential biomarkers for identifying patients at risk for long-term fatigue. However, further research is required to elucidate the specific roles of these biomarkers in post-COVID-19 syndrome and explore their potential as therapeutic targets.

By linking systemic factors such as ICU admission and critical illness to molecular changes in muscle metabolism, this study offers a more comprehensive understanding of the biological mechanisms underlying post-COVID fatigue. This integrative approach underscores the importance of considering both clinical history and molecular biomarkers when assessing long-term post-COVID sequelae, paving the way for more targeted diagnostic and therapeutic strategies.

The lack of significant associations between fatigue and respiratory, cardiac, or standard laboratory parameters underscores the multifactorial nature of post-COVID fatigue. This symptom likely arises from an interplay of physical, psychological, and metabolic factors. While fatigue was associated with poorer health-related quality of life, no consistent link to respiratory or cardiac impairments was identified. This emphasizes the need to investigate non-organ-specific pathways, such as persistent neuroinflammatory or metabolic disturbances, which may contribute to this debilitating condition.

Furthermore, these findings have significant clinical implications. Identifying myostatin and irisin as potential biomarkers paves the way for targeted therapeutic interventions to mitigate muscle dysfunction and fatigue in post-COVID patients. Potential strategies include myostatin inhibitors, structured physical rehabilitation programs, and metabolic modulation therapies. Additionally, this study highlights the critical need for early interventions in ICU survivors to prevent long-term complications associated with muscle deterioration and persistent fatigue.

This study has several limitations. The single-center design may limit the generalizability of the findings. Additionally, the relatively small sample size and reliance on self-reported symptoms introduce the potential for recall bias. The cross-sectional nature of the follow-up assessments restricts the ability to establish causality, particularly regarding the association between ICU admission and persistent fatigue. While elevated levels of myostatin and irisin were observed, the lack of longitudinal biomarker data precludes any analysis of temporal changes in relation to symptom onset and progression. Moreover, data on fatigue remain limited, and no detailed classification of potential subtypes or severity levels has been performed, which could have provided deeper insights into its underlying mechanisms. Despite these limitations, this study offers valuable contributions to understanding the biological foundations of post-COVID fatigue, highlighting the role of muscle-related biomarkers and the impact of critical illness on long-term recovery. By adding to the growing body of evidence on post-COVID sequelae, these findings pave the way for targeted therapeutic strategies and improved clinical management of affected individuals.

## 5 Conclusion

This study offers a novel perspective on the interplay between critical illness, muscle metabolism, and persistent fatigue in post-COVID patients. By identifying key molecular markers and their association with ICU admission, our findings enhance the understanding of post-COVID sequelae and lay the foundation for future research on targeted interventions to improve recovery and quality of life. Future studies should focus on longitudinal assessments of biomarkers and muscle dysfunction over time, mechanistic investigations into ICU-related myopathy and its underlying pathways, expanded multiorgan evaluations to explore broader systemic effects, the role of vaccination in mitigating post-COVID fatigue, targeted rehabilitation strategies to optimize recovery outcomes, and large-scale, multi-center studies to validate findings and enhance clinical applicability.

## Data Availability

The raw data supporting the conclusions of this article will be made available by the authors, without undue reservation.
