# Supplementary material for: Elevated adipokines and myokines are associated with fatigue in long COVID patients
Source: Front Med (Lausanne). 2025 May 19;12:1547886. doi: 10.3389/fmed.2025.1547886 (PMC12127188; doi:10.3389/fmed.2025.1547886)
Supplement: Supplementary file 1 [file Table_1.DOCX]

**Supplementary Table 1**: Median values for lung function parameters in the overall study population and comparison between patients with and without post-COVID-19-fatigue.

| **Variables** | **All patients**  **Md (IQR)** | **Post-COVID fatigue** | |  | |  |
| --- | --- | --- | --- | --- | --- | --- |
|  |  | **Yes**  **Md (IQR)** | **No**  **Md (IQR)** | | **p-value*** | |
| FVC (%P) | 97.5 (85.5 – 111.0) | 100.0 (90.0 –113.5) | 94.0 (85.0 – 101.5) | | 0.137 | |
| FEV1 (%P) | 90.5 (80.2 – 102.0) | 95.0 (84.5 – 107.5) | 88.0 (80.0 – 100.0) | | 0.206 | |
| FEV1/FVC | 76.7 (70.5 – 79.9) | 76.5 (69.2 – 82.0) | 76.9 (71.6 -79.8) | | 0.854 | |
| TLC (%P) | 89.0 (80.0 – 90.0) | 90.5 (84.2 – 102.5) | 88.0 (79.5 – 94.5) | | 0.113 | |
| RV/TLC (%P) | 103.0 (94.0 115.0) | 100.0 (87.2 – 119.2) | 103.0 (96.0 – 114.0) | | 0.379 | |
| DLCO/Hb (%P) | 80.5 (70.7 – 94.0) | 82.0 (74.0 – 96.0) | 80.0 (69.5 – 90.0) | | 0.284 | |
| R5 Hz (%P) | 120.0 (106.0 – 144.0) | 112.0 (99.0 -147.0) | 124.0 (108.0 – 142.7) | | 0.417 | |
| R20 Hz (%P) | 114.0 (99.0 – 139.0) | 112.0 (98.0 134.5) | 115.0 (101.5- 139.2) | | 0.826 | |
| ΔR5 - R20 | 17.1 (11.0 - 26.1) | 15.9 (8.1 – 25.6) | 17.6 (11.9 – 26.1) | | 0.252 | |
| Resonant frequency | 16.2 (13.5 – 22.0) | 16.1 (11.8 – 21.5) | 16.4 (13.9 – 22.2) | | 0.467 | |
| AX | 0.7 (0.5 – 1.6) | 0.6 (0.3 – 1.7) | 0.7 (0.5 – 1.4) | | 0.690 | |
| *****Shapiro-Wilk test; MD, median; IQR, interquartile range; FVC, Forced vital capacity; FEV1, Forced expiratory volume in first second; TLC, Total lung capacity; RV, Residual volume; DLCO, Carbon monoxide diffusing capacity; R5, airway resistance at 5 Hz; R20, airway resistance in 20 Hz; AX, reactance area. | | | | | |  |

**Supplementary Table 2:** Descriptive statistics of SF-36 questionnaire scores stratified by the presence of post-COVID-19-fatigue.

| **Variables** |  | **Post-COVID fatigue** | |  |
| --- | --- | --- | --- | --- |
|  | **All patients**  **Md (IQR)** | **Yes**  **Md (IQR)** | **No**  **Md (IQR)** | **p-value*** |
| Functional capacity | 40.0 (20.0–65.0) | 20.0 (15.0–55.0) | 50.0 (28.7–75.0) | 0.005 |
| Physical aspects | 0.0 (0.0–75.0) | 0.0 (0.0–25.0) | 0.0 (0.0–100.0) | 0.223 |
| Pain | 51.0 (31.0–72.0) | 50.0 (31.0–61.0) | 56.5 (30.7–81.0) | 0.246 |
| General health | 50.0 (37.0–72.0) | 30.0 (22.0–47.0) | 53.5 (45.0–80.0) | <0.001 |
| Vitality | 50.0 (35.0–75.0) | 30.0 (15.0–40.0) | 65.0 (45.0–80.0) | <0.001 |
| Social aspects | 62.0 (37.0–100.0) | 37 (25.0–50.0) | 87.0 (50.0–100.0) | <0.001 |
| Emotional aspect | 0.0 (0.0–100.0) | 0 .0 (0.0–0.0) | 50 (0.0–100.0) | 0.001 |
| Mental health | 64.0 (48.0–80.0) | 52.0 (32.0–64.0) | 76.5 (63.0–88.0) | <0.001 |
| *Mann-Whitney U test; MD, Median; IQR, interquartile range. | | | | |
