# Supplementary material for: Elevated adipokines and myokines are associated with fatigue in long COVID patients
Source: Front Med (Lausanne). 2025 May 19;12:1547886. doi: 10.3389/fmed.2025.1547886 (PMC12127188; doi:10.3389/fmed.2025.1547886)
Supplement: Supplementary file 2 [file Data_Sheet_1.DOCX]

Supplementary Material

# Supplement 1

**Follow-up Clinical Evaluation Questionnaire**

1. Symptoms (consider present if developed or worsened after COVID-19):

- Dyspnea: ( ) Yes ( ) No mMRC ___
- Cough: ( ) Yes ( ) No
- Chest pain: ( ) Yes ( ) No
- Myalgia: ( ) Yes ( ) No
- Fatigue: ( ) Yes ( ) No
- Muscle weakness: ( ) Yes ( ) No
- Arthralgia: ( ) Yes ( ) No
- Headache: ( ) Yes ( ) No
- Memory loss: ( ) Yes ( ) No
- Hair loss: ( ) Yes ( ) No
- Insomnia: ( ) Yes ( ) No
- Anosmia: ( ) Yes ( ) No
- Anxiety symptoms: ( ) Yes ( ) No

2. Return to work activities: ( ) Yes ( ) No ( ) Not applicable

3. Regular exercise (at least 3 times a week): ( ) Yes ( ) No

4. Clinical complications after hospital discharge: ( ) Yes ( ) No

- Deep vein thrombosis: ( ) Yes ( ) No
- Pulmonary embolism: ( ) Yes ( ) No

- Cerebrovascular complications: ( ) Yes ( ) No

- Acute coronary syndrome: ( ) Yes ( ) No

- Neurologic complications: ( ) Yes ( ) No Describe: ______________________

- Other complication: ( ) Yes ( ) No Describe: ___________________________
