# Supplementary figures and images for: Elevated adipokines and myokines are associated with fatigue in long COVID patients
Source: Front Med (Lausanne). 2025 May 19;12:1547886. doi: 10.3389/fmed.2025.1547886 (PMC12127188; doi:10.3389/fmed.2025.1547886)

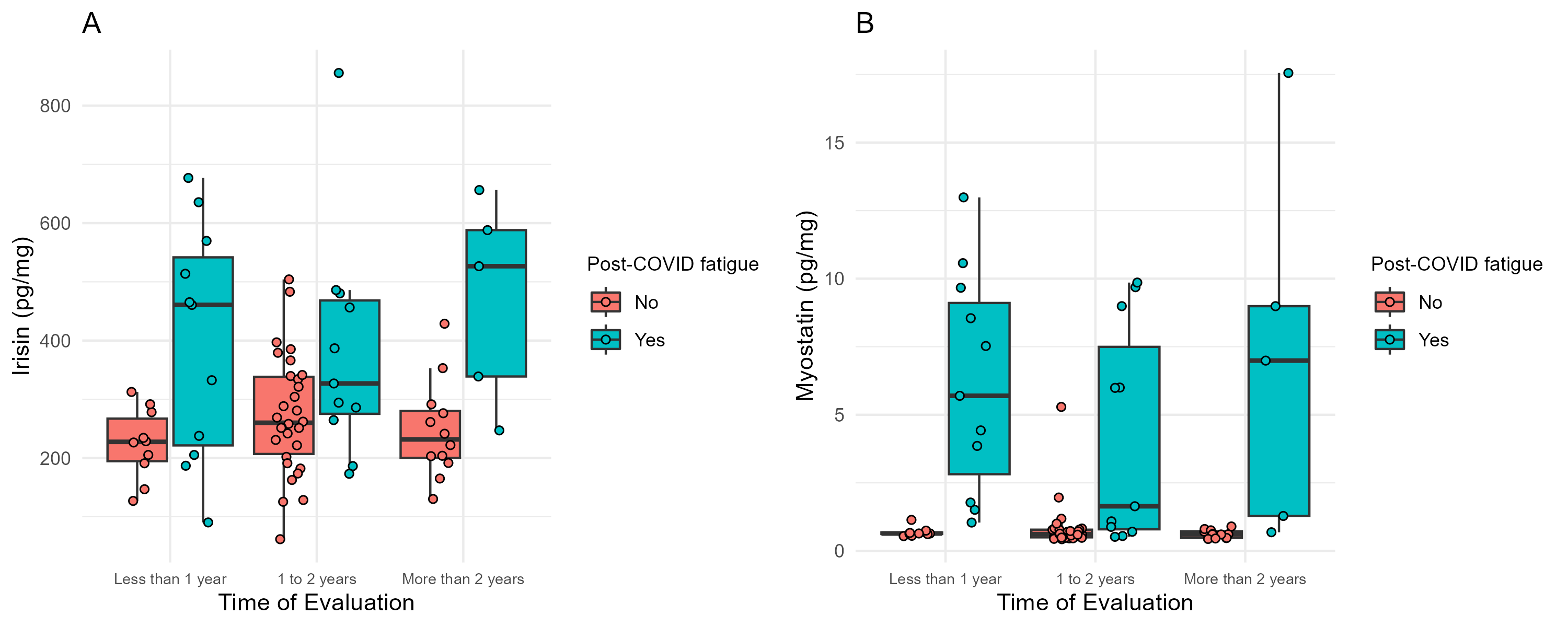

Supplement: Supplementary file 3 [file Image_1.png]

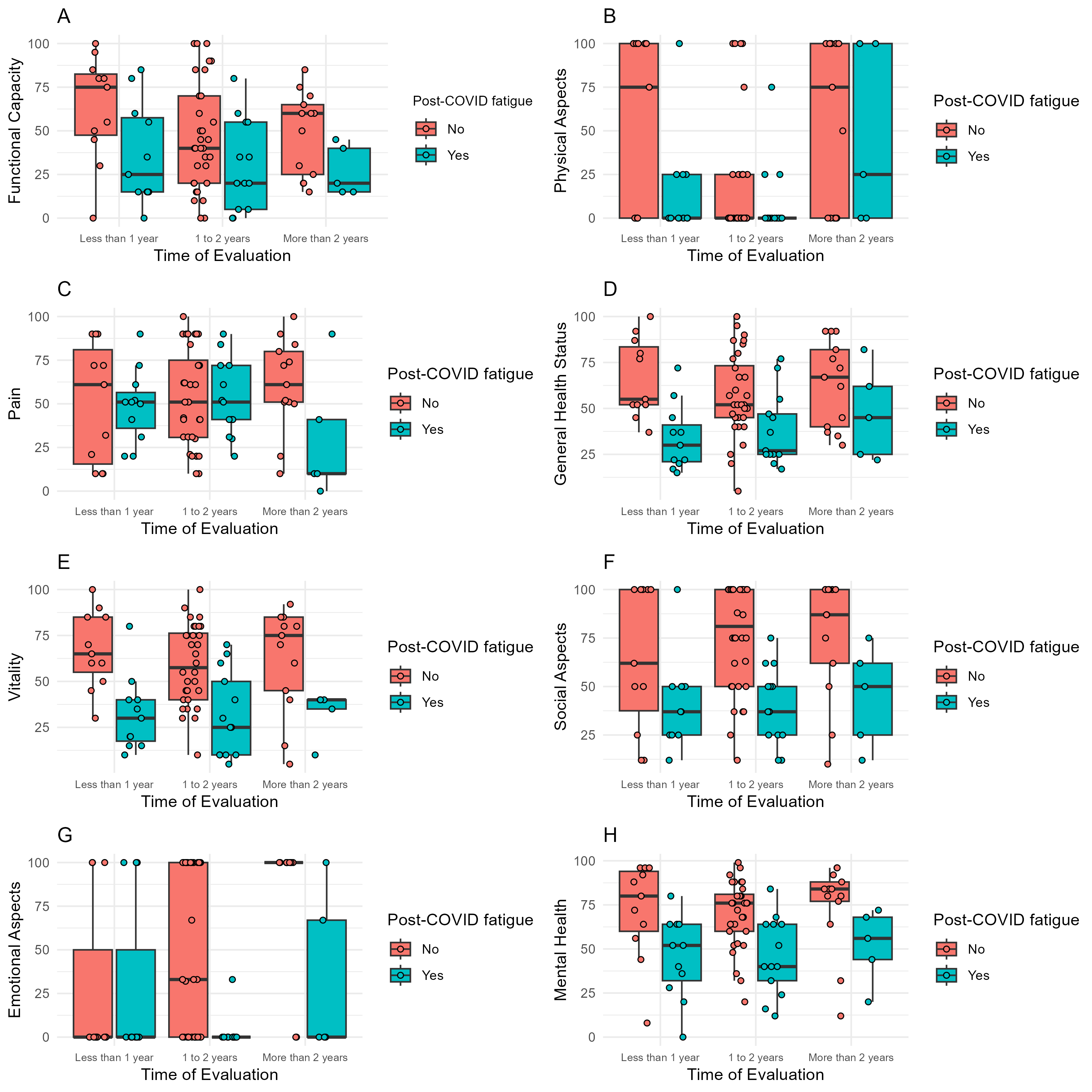

Supplement: Supplementary file 4 [file Image_2.png]

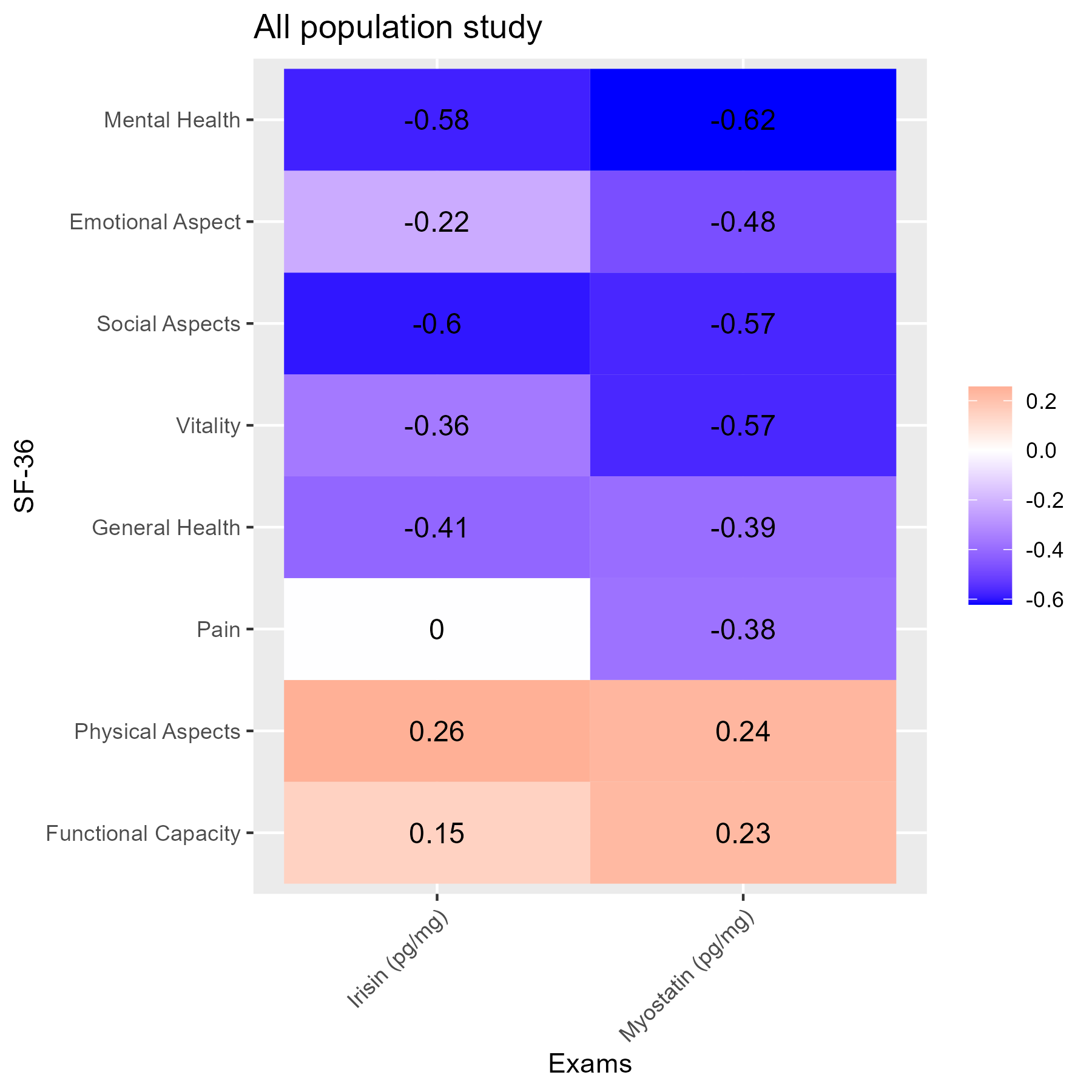

Supplement: Supplementary file 5 [file Image_3.png]

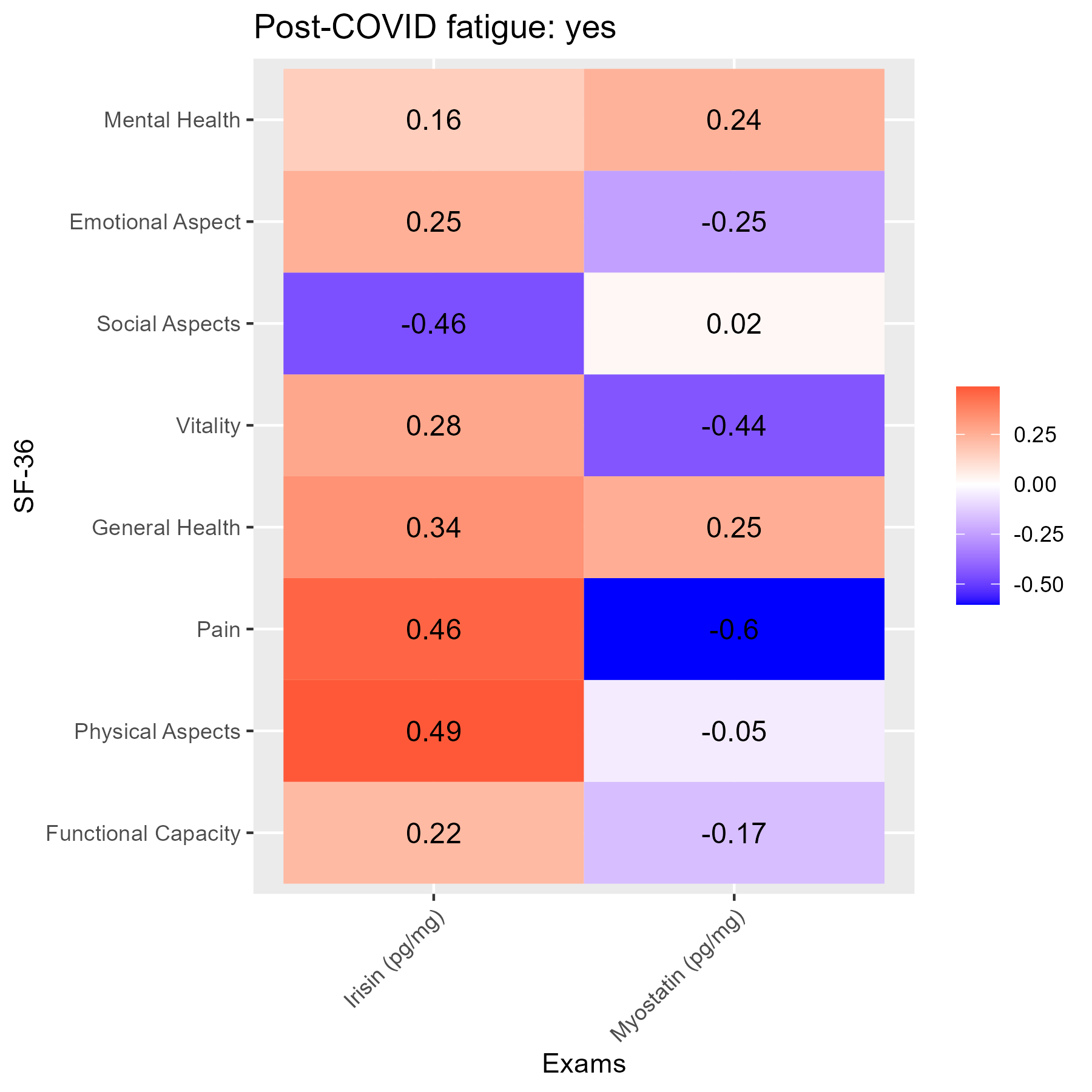

Supplement: Supplementary file 6 [file Image_4.png]

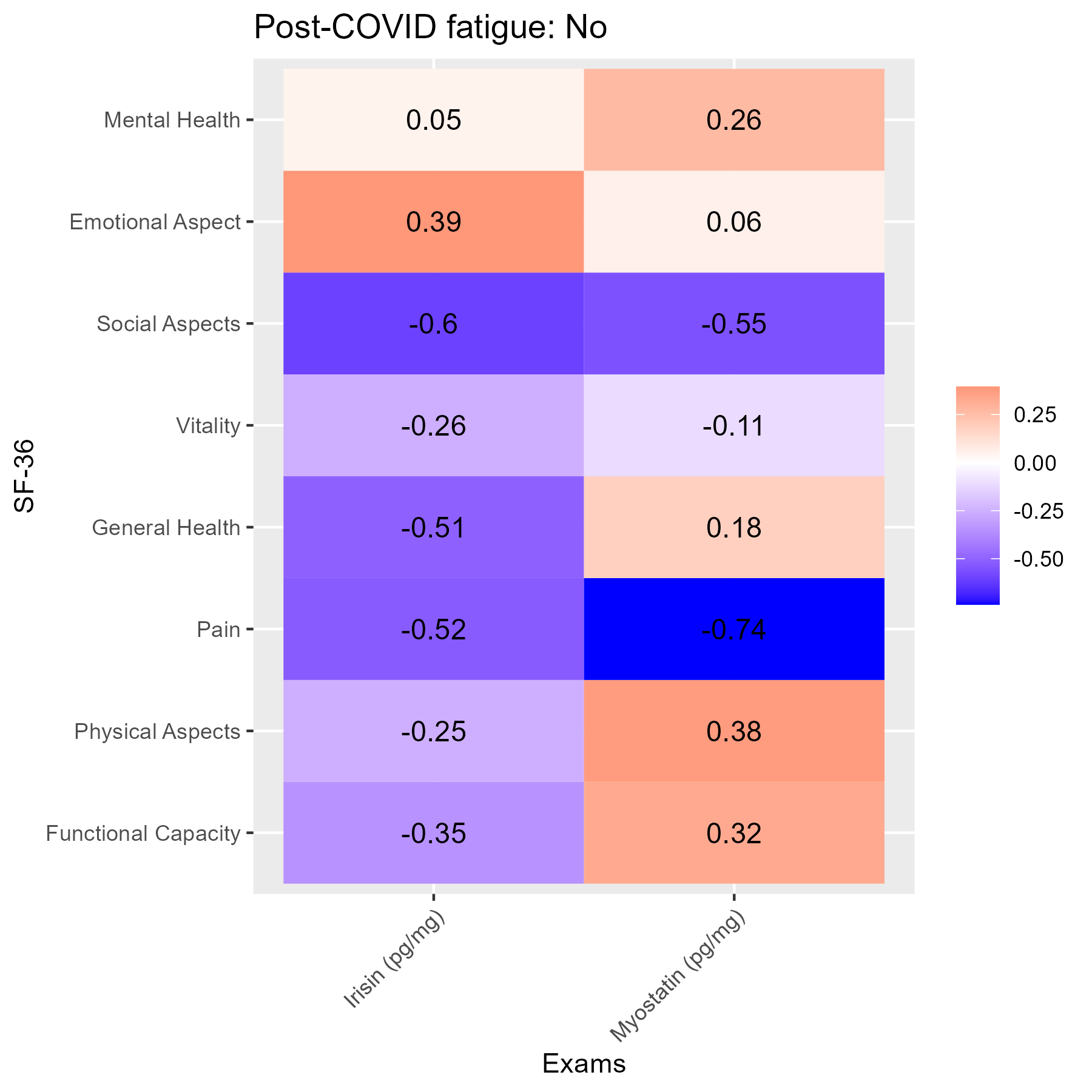

Supplement: Supplementary file 7 [file Image_5.png]
